# Challenges and Opportunities in Building and Maintaining a Good Therapeutic Relationship in Acute Psychiatric Settings: A Narrative Review

**DOI:** 10.3389/fpsyt.2019.00965

**Published:** 2020-01-15

**Authors:** Julia Bolsinger, Matthias Jaeger, Paul Hoff, Anastasia Theodoridou

**Affiliations:** ^1^ Department of Psychiatry, Psychotherapy and Psychosomatics, Psychiatric University Hospital Zurich, Zurich, Switzerland; ^2^ Department of Adult Psychiatry, Psychiatrie Baselland, Liestal, Switzerland

**Keywords:** therapeutic relationship, psychiatry, emergency, acute, coercion, involuntary admission, closed ward

## Abstract

**Background:** The therapeutic relationship and its importance for psychotherapy outcome have been the subject of extensive research over the last decades. An acute psychiatric inpatient setting is a unique environment where severely ill patients receive intensive treatment over a limited, relatively short, period of time. This renders establishing a good therapeutic relationship difficult for various reasons. It seems likely, however, that the therapeutic relationship in such a setting plays a vital role on factors such as clinical outcome, patient satisfaction, and rehospitalization rates. Little information is available on special attributes and caveats of building and maintaining a good therapeutic relationship in an acute psychiatric setting, neither on its influence on therapy success.

**Methods:** An extensive systematic literature search was performed using PubMed, science direct, psyc info, and google scholar databases. Keywords used were therapeutic alliance, therapeutic relationship, psychiatry, emergency, acute, coercion, autonomy, involuntary, closed ward. RCTs, observational studies, reviews, meta-analyses, and economic evaluations were included, case reports and opinion papers were excluded. Factors specific to an acute psychiatric setting were identified, and the available information was categorized and analyzed accordingly. The PRISMA statement guidelines were followed closely upon research and preparation of the present review.

**Results:** A total of 48 studies were selected based on their relevance as well as design. They demonstrated that several factors related to setting, patient attributes, staff attributes, admission circumstances, and general situation, render building and maintaining a good therapeutic relationship difficult in an acute psychiatric setting compared to scheduled, long-term therapeutic sessions. The available literature on how to overcome this dilemma is scarce. Interventions involving staff and/or patients have been shown to be effective in terms of relevant outcome parameters.

**Conclusions:** Increasing research efforts, as well as raising awareness and providing specific competencies amongst clinicians and patients in terms of nurturing a good therapeutic relationship in acute settings, are necessary to improve clinical outcome, economic factors, quality of patient care and patient as well as staff satisfaction.

## Introduction

The therapeutic relationship (TR) has been called the “foundation of mental health practice” (1). A vast body of literature emphasizes the importance of the TR in psychiatry. A positive TR has been consistently shown to be associated with better therapy outcomes in terms of clinical improvement, duration of stay, rehospitalization rate, and patient satisfaction [e.g. (2–6)]. On the other hand, a poor therapeutic relationship is associated with negative therapy outcome, as well as secondary negative effects such as increased risk of violence [e.g. (7)].

In various ways, the TR has been a subject of psychiatric research ever since psychotherapeutic work was first conducted. Most research into the subject of TR stems from psychotherapy in a scheduled, non-acute, long-term one-on-one setting, where the concept was first described (8). One review defined three core aspects of TRs: a collaborative nature, an affective bond between patient and therapist, and a mutual ability to agree on goals (9). It is easily concluded that patient-related, therapist-related, and environmental factors could hinder the establishment of all three of these aspects. For example, one would hypothesize that in the case of involuntary admissions into a closed ward, a patient will be less likely to spontaneously engage in a trustful collaboration with the attending physician. An affective bond between someone in an acute psychiatric crisis and the staff happening to be on duty the day that person is admitted into the hospital will likely be harder to establish than between therapists and their clients getting to know each other in a scheduled appointment agreed upon by both parties. It has also been shown that TR quality is equally important to both staff and patients (10). Being in a mental state that requires emergency in-patient treatment can potentially impede core skills needed in order to define and agree on goals, such as decision-making capacity, standing up for one’s own rights, etc. Examining these factors, along with other variables that are likely to cause issues in building and maintaining a stable TR, is crucial in understanding and developing recommendations for clinical practice.

The term “acute psychiatric setting” is relatively unspecific, and it can be defined and understood in a lot of very different ways. In the present review, any environment providing emergency treatment for unscheduled, unforeseen psychiatric conditions is considered an “acute psychiatric setting”. These settings provide several unique features that render building and maintaining a TR difficult compared to regularly scheduled psychotherapeutic care. At the same time, with regard both to the emergency setting itself and to secondary positive or negative implications resulting from success or failure of handling these situations, respectively, health care professionals would be well advised to pay particular attention to nurturing a stable and positive TR in an acute setting. However, data on this subject is scarce. Three core questions emerge from this situation: One, what are the potential pitfalls and special challenges of TR building and maintenance in an acute setting? Two, how can these risks be met by staff (and potentially patients/their environment) in order to avoid negative outcomes? Three, in how far does TR quality affect clinical outcome, rehospitalization rate, patient and staff satisfaction, economic balance, or other relevant factors? The present article aims to shed some light on the above questions using a review of available publications.

## Materials and Methods

A systematic literature review was conducted in web databases (PubMed, science direct, psyc info, and google scholar) between 12/2017 and 11/2018. Keywords used were therapeutic alliance, therapeutic relationship, psychiatry, emergency, acute, coercion, autonomy, involuntary, closed ward. All keywords were used for individual searches, and the keywords “therapeutic alliance” and “therapeutic relationship” were used in a syntax with “AND” and each other keyword, respectively.

RCTs, reviews, meta-analyses, observational studies, opinion papers, and economic evaluations were included, case reports were excluded. No specific time frame for publication was defined. The attached flow diagram (PRISMA-P-guideline-based) (11) gives an overview of the screening, as well as inclusion/exclusion, processes (cf **Supplementary Material, Image 1**). Reasons for exclusion besides not meeting the inclusion criteria were lack of availability of an English full text version, strongly different settings/patient populations (e.g. minors, incarcerated patients), incomparable systems.

Since our study question proved not to lend itself very well to quantitative analysis, we decided to use a narrative approach in the writing process. For this reason, we also decided against an attempt to define the quoted studies in terms of populations, interventions, comparators, outcomes, and study designs (PICOS). Based on the overview of available information gained from the screening process, as well as insights from clinical routine, sub-sections of particular relevance to TR in an acute psychiatric setting were defined. These sections were labelled “Involuntary admission”, “Increased symptom severity”, “Loss of autonomy”, “Coercion”, “Role conflict therapist: help vs. assess”, “Team work, general setting”, “Short duration, lack of continuity”.

Study results referring to the previously selected keywords, as well as results adding relevant information on either of the defined sections, were analyzed. The risk of bias was controlled on a study level by critically assessing the available information and, where applicable, discussing it accordingly. There may be a risk of publication bias across studies or selective reporting of supporting evidence for individual viewpoints/favored interventions within studies, which might provide a confounding factor in the present analysis and should be borne in mind accordingly.

The PRISMA-P statement guidelines were followed closely upon research and preparation of the present review) (11).

## Results

A total of 48 studies were selected based on their relevance as well as design, as presented in the flow diagram. The information obtained from them is organized and presented in the sections described above. An overview of the main findings from each study can be found in the attached results table (cf **Supplementary Material, Table 1**).

### Involuntary Admission

A relatively large proportion of patients receiving treatment in an acute psychiatric setting are admitted through authorities or their treating physician/therapist against their will. A negative association between involuntary admission and the quality of the TR has been repeatedly found (6, 12–16). On the other hand, it has been shown that psychiatric emergencies are handled better if a stable TR is present, enabling less coercive strategies such as “talking down”. Verbal and nonverbal communication skills have been shown to have crucial effects in the context of TR building and maintenance (17). (First) encounters with psychiatric services as experienced in an emergency situation have been described as being predictive for views on psychiatry and for the quality of future TRs (18). In a study assessing patients’ perceptions of undergoing an involuntary treatment order, four out of the six most frequently described themes were related to staff attitudes and behaviors, indicating that understanding patients’ needs and meeting them accordingly can enable the development of a positive TR despite the obstacles posed by involuntary admission (15).

### Increased Symptom Severity

Being in need of emergency treatment, patients in acute psychiatric settings will tend to present with a higher and/or more acute degree of suffering from their symptoms, at least initially. Quality ratings of the TR have been consistently shown to decrease with increasing symptom severity (19–21). This may be additionally aggravated by acute deterioration of clinical state or a sudden change of external factors previous to hospitalization. While symptom severity upon admission is beyond the control of the therapist, raising awareness of its negative implications not only on the clinical state but also on the ability to build and maintain a TR may enable the development of strategies to overcome this obstacle.

### Loss of Autonomy

Autonomy is a crucial concept for both personal dignity of the patient, as well as for the ability and willingness to engage in a TR. One largely acknowledged definition of autonomy states that “Personal autonomy is, at minimum, self-rule that is free from both controlling interference by others and from limitations, such as inadequate understanding, that prevent meaningful choice” (22). Independently of whether or not a patient was admitted into the hospital voluntarily, it can easily be concluded that acute psychiatric wards have some features that in themselves inevitably restrict a patient’s autonomy. Practices such as closed doors, restricted access for visitors, and limited permission to leave the ward in terms of time and/or distance, are likely to meet the criteria for being a “controlling interference” by most definitions. Psychiatric emergencies have been defined as “an acute disturbance of behaviour, thought or mood of a patient which if untreated may lead to harm, either to the individual or to others in the environment” (23). It would again seem likely for either of these conditions to meet the criteria of being a limitation preventing meaningful choice, thus reducing autonomy drastically. This is likely to pose a significant hindrance to building a good TR if not addressed consciously and carefully.

### Coercion

As with involuntary admission, a negative association between perceived coercion and TR quality ratings has been established (12, 16, 20, 24). Perceived coercion, however different definitions of the concept may be, certainly involves a subjective loss of control. Practices of forcing medication on patients, physical or chemical restraint or seclusion without consent obviously meet the criteria for such perceived coercion. Further negative consequences may result, for example in the case of medication being refused as a means of protest against not only the drug itself, but also against the loss of autonomy associated with “giving in” to the prescribing therapist. On the other hand, a positive TR has been shown to be a predictor for medication adherence in schizophrenic patients (25–27). Therapists would be well advised to make use of this effect in their efforts to provide patients with the best possible treatment, besides the obvious interpersonal benefits.

Depending on professionals’ attitude on coercion, they have been demonstrated to under- or overestimate the extent to which measures are perceived as coercive by patients, respectively (28). Both the presence of coercion in itself and the misjudgment of health care professionals are factors to be addressed in an effort to improve TR building in acute settings.

### Role Conflict Therapist: Help vs. Assess

Bearing in mind their responsibility towards the safety and wellbeing of their patients, therapists can run into conflicting requirements negatively impacting a trustful TR. For example, in patients with acute suicidal ideations, a therapist may, despite aiming to be as transparent and open as possible, choose not to disclose additionally disturbing information they have received through third parties (death of a relative, spouse’s wish to divorce, etc). Equally, information that may provoke aggression may be withheld from acutely agitated patients, in line with the previously described principle of prioritizing safety over the TR in specific situations (29). When it comes to applying coercive measures or to evaluating a patient’s decision-making capacities, therapists may have to make decisions that they consider to be in their patients’ best interest from a medical-professional point of view, yet thus endanger the TR if the patient disagrees with this judgement. Ensuring either the patient’s, the therapist’s or third parties’ safety can also require taking measures that are likely to decrease the TR quality (30). While it has been demonstrated that interpersonal fairness improves TR and compliance (31), the above examples are likely to reduce the perceived degree of fairness on the therapist’s part. Sensitive handling of both deciding in these situations and communicating the respective decisions requires adequate training in order to still maintain a good TR.

### Team Work, General Setting

The management of acute psychiatric patients is performed by an interdisciplinary team. Professions other than the principal therapist face their own difficulties in building and maintaining a TR in this setting. This has been described most predominantly in the nursing profession [e.g. (32)]. Difficulties in the relationship with one person or profession can have a “spillover-effect”, burdening the TR between patient and principal therapist, or they can further conflicts in the multiprofessional team. Some patients may also find it more difficult to engage in a TR with a therapist working in a team (as opposed to seeing an individual therapist in an outpatient practice), feeling intimidated by the loss of privacy following the necessary exchange between team members. Due to the organization of acute wards with shift systems, responsibilities may change, with patients finding themselves in contact with unfamiliar staff repeatedly. This will certainly decrease the probability of establishing a trustful, high-quality TR.

Patients’ preferences regarding the gender, personality, background or other attributes of their therapist can hardly be taken into account in an acute psychiatric setting. It has been shown that factors such as communication, cultural sensitivity, and building a TR in an individualized way are pivotal to a good TR and to feeling safe in a therapeutic milieu (24, 33, 34). Bearing this in mind and making conscious efforts to overcome these hindering conditions and meet an individual patient’s needs would be well served in facilitating the building of a good TR.

### Short Duration, Lack of Continuity

Acute in-patient treatment tends to be short, and decisions may be required to be taken very early after the first encounter between patient and therapist. For example, if an acutely psychotic patient is admitted involuntarily, accompanied by state authorities, the very first interaction between therapist and patient may take place in the presence of these authorities and in the context of coercive measures. Aside from the other difficulties associated with such a setting, both the time to build and to maintain a good TR are thus condensed immensely compared to elective psychotherapy. Besides that, there is usually not a perspective for the patient and the therapist to continue their TR after the patient is discharged. Knowing the temporal limitations of the TR may reduce the level of trust in the therapist that a patient is willing to invest, and likewise may diminish a therapist’s readiness to engage in earning that trust.

## Discussion

Several points become evident from the above considerations. First, it would seem highly advisable to expand efforts to specifically adapt research methods to examining the TR in an acute setting as opposed to struggling with unsuccessful adaptations of TR research from psychotherapy settings. Most of the above sections defined as influential for TR in an acute setting are not applicable to TR in a psychotherapeutic setting, and thus require research methods more specifically tailored to examine factors related to these sections and their influence on the TR. The need for an adaptation of methods has been pointed out previously (8). Certain efforts have been made to develop scales to objectively measure TR-related factors (35). It has been pointed out that furthering and developing such measures, preferably in a standardized way, would seem reasonable (36).

An association between patient satisfaction and TR quality ratings has been shown (37–39). Aiming to gain a better understanding of the peculiarities and potential pitfalls of building and maintaining a good TR in an acute setting should thus involve patient-focused research, e.g. through satisfaction questionnaires (40, 41), although receiving feedback from patients in severe distress may require some adaptation of standard processes (42). It has been pointed out that a dialog between patients and therapists/staff on delicate subjects such as coercion is possible, useful and desired by patients (43). Establishing transparent communication on these subjects could diminish the probability of misunderstanding and enable more individualized treatment options meeting patients’ needs more adequately. It seems plausible to hypothesize that such measures would thus improve the quality of the TR.

In certain patient forums, there are efforts to protect the TR from a patient’s perspective for example (44). Internet-based opportunities for exchange and assessment will likely increase, and it has been shown that a TR develops even when there is no direct face-to-face contact (45). Making these efforts known amongst therapists, as well as supporting similar initiatives in different communities, would certainly raise awareness and have beneficial effects on TR quality. Such initiatives should take into account both general factors, those specific to an acute setting, as well as potential diagnosis-related pitfalls (e.g., paranoid fears in schizophrenia, narcissistic wound in personality disorders, lack of motivation in depression, etc). Furthering exchange between therapists and patients might help overcome misunderstandings, as well as reduce the frequently observed disparity between their respective ratings of TR quality [e.g. (46–49)].

It has been pointed out that therapists, on the other hand, should receive specific training (3), for example in verbal and nonverbal communication skills (8, 17, 50) and that awareness of difficult circumstances and situations should be raised, including other professions such as nurses (50). Recommendations have been made for therapists to minimize perceived coercion and to mediate procedural fairness when attempting to improve patients’ treatment adherence (28), which would likely have a positive influence on TR quality, as well. One study observed that the quality of the TR was significantly worse in acute wards than crisis houses, suggesting the latter as an alternative model for suitable patients (51). Examining the reasons for the difference in quality ratings might further therapists’ understanding of potential improvements to TR building in an acute ward. Another important development in the management of psychiatric emergencies is the establishment of crisis intervention teams visiting clients in their homes (52). Research on therapeutic relationship in these interventions is scarce, but client satisfaction surveys suggest an overall positive perception, which might point towards positive interactions and thus a good TR in such settings. Further examining the methods used in those home treatment interventions might provide useful insights for building a good TR in acute wards, as well as shedding some light on the preferred mode of intervention in various crisis settings. Additionally, environmental factors such as family and other social contacts might be better accounted for and given a chance to participate in the therapeutic process in more outpatient-directed approaches.

The scarcity of available data on some of the described factors, as well as the paucity of specific research into TR in acute psychiatric settings, also lead to limitations of the present review.

## Conclusion

As a conclusion, efforts to raise awareness of potential pitfalls in building and maintaining a good TR in an acute setting, conducting more specific research into this area, questioning patients and including them in the process of decision-making wherever possible, defining standardized instruments as well as enabling more specifically tailored procedures, and developing training options for therapists based on these findings would likely increase the chances of improving TR quality in acute psychiatric wards.

Implementing the above measures would likely have positive impacts not only on clinical outcome, duration of stay, rehospitalization rate, and patient satisfaction, but also on experience with and perception of psychiatry, possibly paving the way for a smoother course of disease and treatment in the case of chronic illness as well as reducing stigma.

## Author Contributions

JB and AT have made substantial contributions to conception and design of the study. JB executed the acquisition of data. JB and AT analyzed the data. JB, PH, MJ, and AT have been involved in the interpretation of data, drafting, and revising the manuscript critically for important intellectual content. All authors read and approved the final manuscript.

## Conflict of Interest

The authors declare that the research was conducted in the absence of any commercial or financial relationships that could be construed as a potential conflict of interest.

The reviewer MZ declared a past co-authorship with one of the authors MJ to the handling editor.

## Abbreviations

TR, Therapeutic relationship.

## Supplementary Materials

The Supplementary Material for this article can be found online at: https://www.frontiersin.org/articles/10.3389/fpsyt.2019.00965/full#supplementary-material

Supplementary Image 1 Flow diagram.

Supplementary Table 1 Results table.
