# Supplementary material for: Challenges and Opportunities in Building and Maintaining a Good Therapeutic Relationship in Acute Psychiatric Settings: A Narrative Review
Source: Front Psychiatry. 2020 Jan 15;10:965. doi: 10.3389/fpsyt.2019.00965 (PMC6974619; doi:10.3389/fpsyt.2019.00965)
Supplement: Supplementary Image 1 — Flow diagram. [file Image_1.pdf]

Flow Diagram (following PRISMA guidelines)

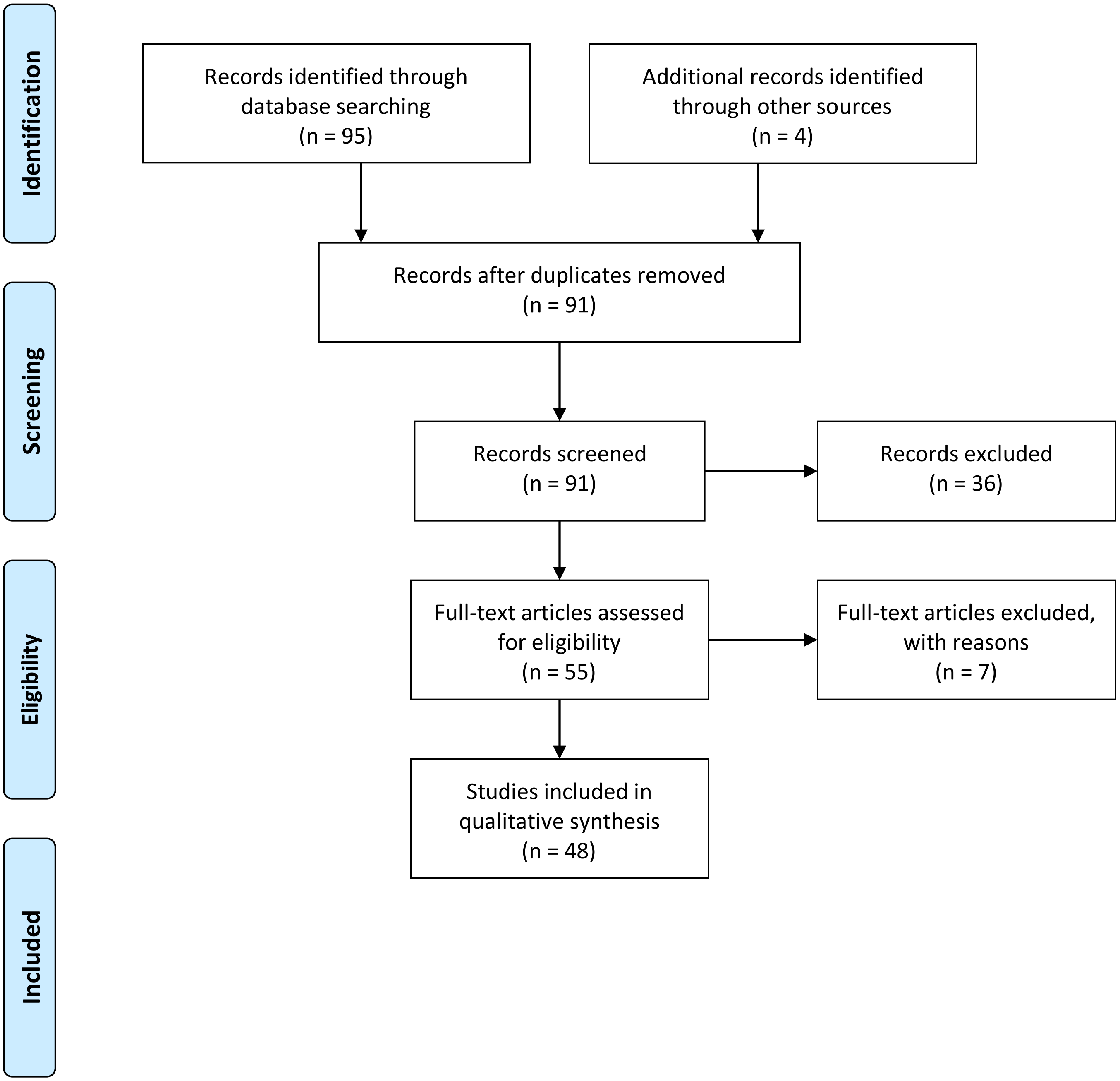

From: Moher D, Liberati A, Tetzlaff J, Altman DG, The PRISMA Group (2009). *P*referred *R*eporting *I*tems for *S*ystematic Reviews and *M*eta-*A*nalyses: The PRISMA Statement. PLoS Med 6(7): e1000097. doi:10.1371/journal.pmed1000097

For more information, visit www.prisma-statement.org.
